# Nonresponsive Parenting Feeding Styles and Practices and Risk of Overweight and Obesity among Chinese Children Living Outside Mainland China: An Integrative Review of the Literature

**DOI:** 10.3390/ijerph20054090

**Published:** 2023-02-24

**Authors:** Qun Le, Mary L. Greaney, Ana Cristina Lindsay

**Affiliations:** 1 Department of Public Health, University of Massachusetts Lowell, Lowell, MA 01854, USA; 2 Department of Health Studies, University of Rhode Island, Kingston, RI 02881, USA; 3 Department of Exercise and Health Sciences, University of Massachusetts Boston, Boston, MA 02125, USA

**Keywords:** parenting feeding styles, parenting feeding practices, Chinese, immigrants, children

## Abstract

Chinese immigrants are a fast-growing population group in many parts of the world. Childhood obesity is increasingly a public health problem among Chinese living outside mainland China. Evidence suggests that parenting feeding styles and practices critically influence children’s eating behaviors and risk of being overweight or obese. Therefore, the objectives of this review were to identify and synthesize the evidence from studies examining the associations between parenting feeding styles, feeding practices, and risk of overweight and obesity among children of Chinese parents outside mainland China. A systematic search of four electronic databases (CINAHL, Medline, PsycINFO, and PubMed) was conducted to identify peer-reviewed studies published in English between January 2000 and March 2022. Fifteen studies met the inclusion criteria and were included in the review. Findings of some of the reviewed studies showed that parenting feeding styles and practices varied according to children’s age, gender, weight, and parents’ acculturation levels. The two most identified parenting feeding styles were indulgent and authoritarian. Parents classified as having indulgent and/or authoritarian feeding styles reported using several feeding practices with unintended detrimental effects, such as pressuring children to eat and restricting or controlling child food intake (type and amount). Some of these feeding practices were associated with an increased risk of child overweight. The findings of this review provide important information that can inform the design interventions to address modifiable nonresponisve parental feeding practices such as pressuring, restricting, and controlling that meet the specific needs of Chinese parents and children outside mainland China.

## 1. Introduction

Chinese immigrants are a fast-growing population group in several parts of the world, including the United States (U.S.), Canada, Australia, Europe, and Southeast Asia. In the U.S., for example, the Chinese immigrant population increased by 40.0% from 2000 to 2010 [1]. Evidence suggests that with acculturation, the process of adapting to a new culture and living environment, changes in Chinese immigrants’ dietary habits and physical activity patterns occur [2,3]. These changes may contribute to increases in the rates of overweight and obesity among Chinese populations outside mainland China [4,5,6]. Studies published between 2002 and 2006 show that the prevalence of childhood overweight and obesity was lower (17.4%) among children ages six to nine in China than among in Chinese-American children (31%) in the U.S. [4,5,6]. Furthermore, research demonstrates that with the same body mass index (BMI), Asian children and adults have a higher body fat percentage than non-Hispanic Whites, Hispanic, and Black peers [7,8,9]. Moreover, evidence indicates that Chinese immigrants are at higher risk of developing hypertension, cardiovascular diseases, type 2 diabetes mellitus, and dyslipidemia than European decedents with the same BMI [9,10].

A growing body of literature documents parents’ critical influence on their children’s eating behaviors and, ultimately, weight status [11,12]. Parents influence their children’s development and maintenance of eating behaviors and weight status through their parenting feeding styles and practices [13,14], which affect children’s innate ability to self-regulate food intake, eating behaviors, and their risk of overweight and obesity [11,12].

Parenting feeding styles encompass the overarching attitudes and behaviors that characterize how parents interact with their children across the general parenting domain based on their responsiveness and demandingness [15]. Responsiveness is parents’ degree of warmth, while demandingness refers to their degree of control [15]. Responsiveness and demandingness have been used to identify four parenting style typologies: (1) authoritative (high responsiveness/warmth and high demandingness/control), (2) authoritarian (low responsiveness/warmth and high demandingness/control), (3) indulgent (high responsiveness/warmth and low demandingness/control), and (4) uninvolved (low responsiveness/warmth and low demandingness/control [13,16,17]. Moreover, parenting feeding styles and practices vary according to parents’ cultural background [13,17,18].

Parenting feeding practices are the behaviors that characterize parents’ interactions with their children and the beliefs and attitudes underpinning these interactions [19,20,21,22]. More specifically, parenting feeding practices are behaviors that parents use to directly influence the type and amounts of foods their children consume, including modeling, pressuring, restricting, monitoring, rewarding, etc. [13,23,24].

Recently, several studies in mainland China have examined feeding styles and practices [25,26,27,28,29]. These studies found that caregivers of children ages three to five used authoritarian and indulgent feeding styles most frequently. They also revealed that mothers are more likely to have authoritarian feeding styles than fathers. At the same time, fathers with higher education are more likely to have authoritarian and permissive feeding styles, while grandparents are more likely to have a permissive feeding style [25]. Furthermore, mothers’ authoritarian feeding style was associated with pre-school children having a healthy BMI [25,26,27,28,29]. In contrast, grandparents’ indulgent feeding style was associated with high BMI pre-school children [27]. Studies examining parenting feeding practices [26,28,29,30] have found that Chinese parents’ beliefs and attitudes influence their use of several nonresponisve parenting feeding practices, such as restriction, monitoring, and pressuring children to eat with the intent to encourage and discourage certain foods or the amount their children consume [26,28,29]. Research indicates these nonresponsive feeding practices are associated with an increased risk of being overweight among pre-school-age children [30].

Furthermore, research has also found differences in parenting styles and feeding practices between Chinese immigrants and Chinese parents in mainland China [31,32,33,34,35]. Studies suggest that parenting feeding styles and practices vary by length of time since immigration, countries of immigration, and parents’ acculturation level [31,32,33,34,35]. For example, among recent Chinese immigrants living in England, parents with low acculturation levels were more likely to have indulgent feeding styles than parents with higher acculturation levels [35]. In contrast, a study conducted among Chinese immigrant mothers of school-age children living in New York City, U.S. found that mothers with high acculturation levels used indulgent and authoritarian feeding styles, while mothers with low acculturation levels used authoritarian feeding styles [34]. Similarly, Chinese immigrant mothers with school-age children living in California, U.S., classified as having an authoritarian parenting feeding style, had high acculturation levels [31]. Additionally, studies examining parenting feeding practices among Chinese immigrant mothers of school-age children found that they were more likely to use restriction and pressure children to eat than non-Hispanic White mothers [32,34]. In addition, pressuring children to eat was found to be more common among Chinese immigrant parents living in the U.S. with low levels of acculturation than those with higher levels of acculturation [33,34].

Given the increasing prevalence of childhood obesity among Chinese immigrant populations outside mainland China, the influence of parents’ acculturation levels on parenting feeding styles and practices, and the influence of feeding styles and practices on children’s eating behaviors and risk of overweight and obesity, the objectives of this review were to (1) identify and synthesize existing research examining associations between parenting feeding styles and practices and risk of overweight and obesity among Chinese parents of children 2–12 years of age living outside mainland China, (2) highlight the limitations of reviewed studies, and (3) generate suggestions for future research.

## 2. Materials and Methods

Using guidelines developed by Whittemore and Knafl (2005) [36], this review included qualitative, quantitative, and mixed methods studies. The review followed five steps: (1) problem identification, (2) literature search, (3) data evaluation, (4) data analysis, and (5) presentation. In addition, the reporting guidelines of the Preferred Reporting Items for Systematic Reviews and Meta-Analysis (PRISMA) statement [37], which include a four-phase flow diagram, guided the inclusion and exclusion of studies.

### 2.1. Search Strategy

Electronic databases were searched, including the Cumulative Index to Nursing and Allied Health Literature (CINAHL), Medline, PsycINFO, and PubMed, between December 2020 and March 2022. The search was limited to full-text, peer-reviewed manuscripts published in English between January 2000 and March 2022. Search terms included: (1) child OR children OR preschool OR adolescent; (2) ‘feeding practice’ OR ‘feeding behavior’ OR ‘feeding strategy’ OR ‘feeding style’; (3) parent OR caregiver; (4) immigrant OR immigration; and (5) Chinese OR China.

**Figure 1:**
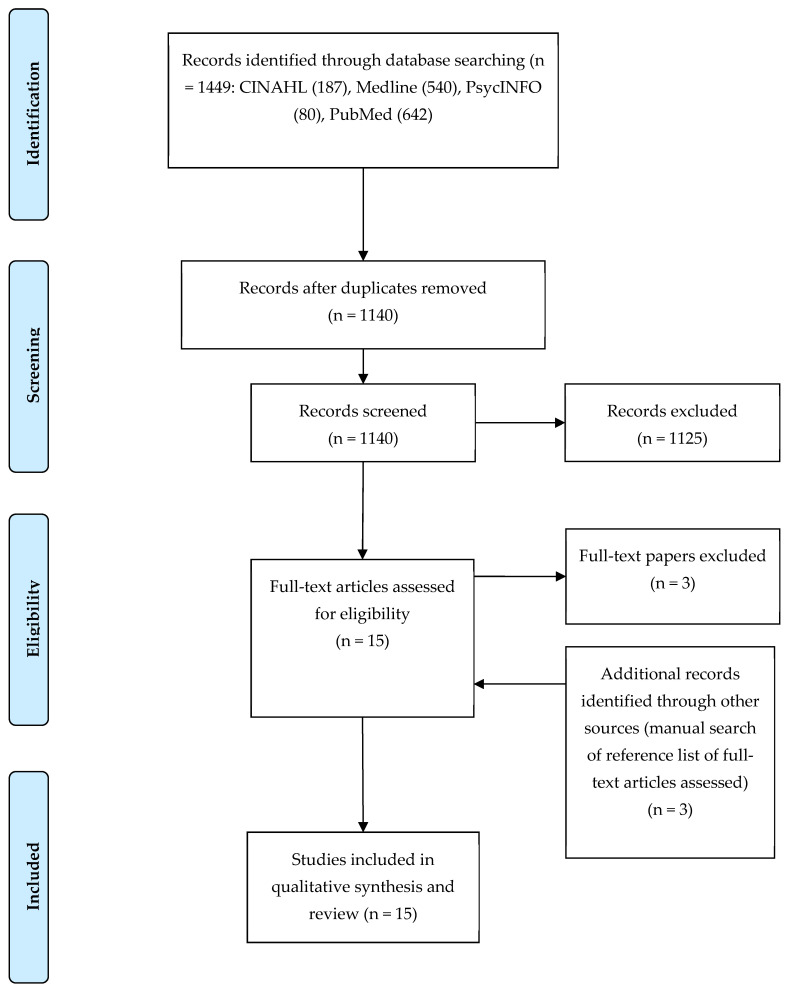
PRISMA 2009 flow diagram. From: Moher D, Liberati A, Tetzlaff J, Altman DG, The PRISMA Group (2009). Preferred Reporting Items for Systematic Reviews and Meta-Analyses: The PRISMA Statement. PLoS Med 6(7): e1000097. doi:10.1371/journal.pmed1000097.

Two authors independently examined the titles and abstracts of all identified citations (QL, ACL). Studies were excluded when both authors determined that the study did not meet the inclusion criteria. The same two authors then reviewed the full text of articles of possibly eligible studies separately to confirm study eligibility further. In addition, these two authors searched the reference lists of the full articles meeting inclusion criteria to identify additional possible studies. Finally, two authors examined and agreed upon the final sets of eligible articles. The search strategy using the PRISMA flow diagram is illustrated in Figure 1 (supplementary material).

### 2.2. Study Selection

This review was limited to studies of normally developing children (i.e., not born preterm, not diagnosed with any physical or mental complications, etc.) of Chinese immigrants living outside mainland China. Qualitative and quantitative studies were eligible for inclusion if: (1) they were peer-reviewed, full-text articles published in English between January 2000 and March 2022; (2) the sample included Chinese immigrant parents (18+ years old) living outside of mainland China; and (3) in the case of studies with multi-ethnic samples, at least 20% of the total sample was Chinese-born immigrant mothers.

### 2.3. Data Extraction and Data Synthesis

All eligible studies were analyzed and synthesized using the Matrix Method [38]. Two authors (QL, ACL) independently read all articles and completed a data extraction form for each article. The form gathered the following information: (1) authors; (2) year of publication; (3) location of study (countries/regions); (4) study population(s); (5) study design; (6) study aims; (7) measures of feeding practices; and (8) study findings. The two authors then compared the completed data extraction forms. A third author (MLG) gave feedback to resolve discrepancies. Due to the inclusion of studies using qualitative and quantitative designs, conducting a meta-analysis was not appropriate. Therefore, the results of this review are presented as a narrative summary.

### 2.4. Quality Assessment of Included Studies

Included studies were evaluated using two quality frameworks: the Strengthening in the Reporting of Observational Studies in Epidemiology (STROBE) guidelines and the Critical Appraisal Skills Program (CASP). Using the STROBE guidelines [39], two authors (QL, ACL) independently assessed the quantitative studies (*n* = 14) for possible bias and methodological issues using a quality checklist created for this review. The checklist consisted of nine questions (see Table 1) designed to be answered with either ‘no’ or ‘yes.’ A score of 0 was given to each ‘no’ response, while 1 point was assigned to each ‘yes’ answer. Scores were summed (possible range of 0–9), with each study being rated. Studies were rated as strong (score > 7), moderate (score 6–7), or weak (score < 6). The one qualitative study included in this review was assessed using the CASP [40], a nine-question appraisal tool (see Table 2), which was completed independently by two researchers (QL, ACL) who discussed and resolved any differences in scoring (Table 1).

## 3. Results

### 3.1. Search

As shown in Figure 1, the search strategy generated 1140 unique articles. Of these, 1125 were excluded for not meeting the eligibility criteria, and 15 full-text articles were selected for detailed review. Of these 15 articles, three were excluded after the detailed review. Additionally, three more articles were identified through a manual search of the reference list of the full-text articles selected for detailed review and assessment. Fifteen research articles [31,32,34,41,42,43,44,45,46,47,48,49,50,51,52] were deemed eligible and included in this review.

### 3.2. Summary of Included Studies

Included studies fell into three categories: (1) studies that included parents of pres-chool-age children only, (2) studies that included parents of school-age children only, and (3) studies that included parents of both pre-school-age and school-age children. Of the 15 included studies, two focused on feeding styles [31,49], eight on feeding practices [32,42,44,46,47,48,50,52], and five on both [34,41,43,45,51].

The 15 studies took place in five countries or regions—nine in the U.S., three in Hong Kong, one in Australia, one in Taiwan, and one in the United Kingdom (U.K.). Fourteen employed quantitative methods, 11 of which were cross-sectional [31,32,34,41,42,43,44,45,46,47,48]; two were longitudinal [49,50,51]; and one was a pilot intervention study [50]. Only one study employed qualitative methods (focus group discussions) [52] (see Table 3). Sample sizes ranged from 22 to 4553 participants (see Table 4). Moreover, the majority of studies (9/14) included only mothers [31,32,34,41,46,48,50,51,52], while six studies included both mothers and fathers [42,43,44,45,47,49] (see Table 4).

### 3.3. Studies of Pre-School-Age Children Only

Seven studies that examined the relationships between parenting feeding styles, feeding practices, and the weight status of pre-school-age children were included in this review. Parents in these studies had children between the ages of one and six years [43,44,46,47,49,50,52] (see Table 4).

#### 3.3.1. Parenting Feeding Styles

One study examined the association between parenting feeding styles of pre-school-age children in Hong Kong [49]. Using a two-wave longitudinal design, this study determined that more permissive and authoritarian parenting feeding styles were associated with less healthy family feeding practices (e.g., allowing unhealthy food, having more relaxed meal routines) and children having more eating behavioral problems [49]. In addition, family feeding practice was a mediator between permissive/authoritarian parenting and frequency of child behavioral problems.

#### 3.3.2. Parenting Feeding Practices

Seven studies explored parent–child feeding practices [43,44,46,47,49,50,52]. In addition, three of the seven studies also examined parents’ perception of responsibility for child feeding, parent weight, child weight, and concern about child overweight [32,46,50]. Of the seven studies, only one used a qualitative design. This study identified four culturally emphasized feeding practices: regulating healthy routines and food energy, spoon-feeding/chasing after, using social comparison to pressure the child to eat, and making an effort to prepare/cook specific foods among immigrant mothers in the U.S. [52].

A pilot intervention study conducted in Hong Kong with Chinese immigrant fathers and mothers of pre-school-age children found that more supervision of children’s eating was positively associated with children’s higher z-BMI. In contrast, restrictive feeding was not associated with children’s weight status [50]. A cross-sectional study conducted in the U.S. found that mothers pressuring children to eat was negatively related to children’s weight status [44].

A cross-sectional study conducted among new Chinese immigrant mothers in Australia found that maternal use of food as a reward, concern about children’s weight, restrictive feeding, and perception of responsibility for cooking were not related to children’s weight status [46]. Furthermore, study findings showed that mothers pressuring children to eat was influenced by the child’s gender, with mothers being less likely to pressure girls to eat than boys. This difference was particularly noticeable when mothers perceived their daughters as being overweight. In contrast, pressuring children to eat was common among parents of boys regardless of parents’ perceptions of boys’ weight status [46].

Furthermore, a few studies found that parents use of parenting feeding practices varied by food groups [47,52]. For example, one study found that parents ‘prompting and encouraging’ children to eat and ‘controlling over child eating’ were positively associated with higher consumption of fruits, vegetables, and dairy products. Additionally, ‘instrumental feeding’ and ‘emotional feeding’ were related to greater consumption of high-energy-dense foods (e.g., sweets, soft drinks, and sugary drinks), whereas ‘control overeating’ was associated with lower intake of high-energy-dense foods [47].

### 3.4. Studies with School-Age Children Only

Five studies examined the relationships between parenting feeding styles, parenting feeding practices, and children’s weight status (see Table 4). Four of these studies used a cross-sectional study design [31,34,41,45], while one was a longitudinal study [51]. Furthermore, four studies included mothers only, and children’s ages in these studies ranged from five to 11 years [31,34,41,51]. Only one study included mothers and fathers, and children’s ages in this study ranged from five to 12 years [45].

#### 3.4.1. Parenting Feeding Styles

A cross-sectional study conducted in the U.K. examining how maternal feeding styles may differ by ethnicity found that compared to White British and Black British, Chinese mothers were significantly more likely to use authoritarian feeding styles with their children [41]. Similarly, a cross-sectional study in New York, U.S. determined that Chinese immigrant mothers’ two most common feeding styles were indulgent or permissive and authoritarian, respectively [34]. In addition, the study determined that children of mothers classified as having an indulgent feeding style were more likely to be overweight or obese than children of mothers having an authoritative feeding style [34]. Likewise, a cross-sectional study conducted in California, U.S., with Chinese mothers of children 8 to 10 years old found that indulgent parenting feeding style was positively associated with children’s higher BMI [31].

Finally, a longitudinal study in Taiwan found that parenting feeding styles moderated the association between parenting feeding practices [51]. Additionally, mothers with a more authoritarian parenting feeding style were more likely to have an overweight child at one-year follow-up [51].

#### 3.4.2. Parenting Feeding Practices

Four studies [34,41,45,51] examined parenting feeding practices. A cross-sectional study in the U.K. found that Chinese immigrant mothers were more likely than South Asian, White British, and Black British mothers to use restrictive feeding practices [41]. Furthermore, the same study found that Chinese immigrant mothers were more likely to pressure children to eat, and had lower levels of instrumental feeding and emotional feeding than South Asian, White British, and Black British mothers [41]. A cross-sectional study conducted in New York, U.S. found that pressure to eat healthy foods was correlated with a child’s BMI percentile while restricting less healthy foods was not [34]. Similarly, a cross-sectional study conducted in 14 states in the U.S. found that encouragement to eat was associated with lower BMI *z*-scores among school-age children [45]. In addition, the same study found that parents who reported monitoring and restriction were more likely to have girls n with higher BMI *z*-scores, while pressuring to eat was associated with lower BMI *z*-scores among boys [45].

A longitudinal study conducted in Taiwan found that mothers’ perception and concern about missing something was associated with ‘pressure to eat,’ with mothers of underweight children being more likely to pressure their children to eat than mothers of overweight children [51]. After controlling for children’s baseline weight status, mothers who were more authoritative used two feeding practices, concern about child weight and monitoring. These were significant predictors of child overweight status [51]. Children were more likely to be overweight at follow-up if they had a mother who used authoritarian feeding practices. At the same time, children with mothers with authoritative feeding styles were less likely to be classified as overweight at follow-up if their mothers monitored their children’s dietary intake [51].

### 3.5. Studies including Both Pre-School and School-Age Children

Three studies examined the relationship between parenting feeding practices and children’s weight status among both pre-school- and school-age children (see Table 4). Children’s ages in these studies ranged from 2 to 12 years [32,42,48]. Only one of these studies reported findings by age group (i.e., pre-school and school-age) [32]. None examined parenting feeding styles. All three studies included only mothers [32,42,48], even though one study collected demographic information of both mothers and fathers [48].

#### Parenting Feeding Practices

A cross-sectional study in Philadelphia, U.S. found that Chinese immigrant parents reported higher mean scores than non-Hispanic white mothers for concern about their children’s weight status and restricting their children’s access to foods in both age groups (pre-school and school-age) and monitoring in the pre-school group. Among Chinese immigrants, concern about child weight and monitoring of the children’s eating were not associated with the children’s weight status (z-BMI) [32]. However, mothers pressuring to eat was inversely associated with the children’s weight in the school-age group, but not the pre-school group. Furthermore, restriction was positively associated with child z-BMI. However, these associations were not significant in adjusted analyses [32].

A cross-sectional study conducted in Maryland, U.S. with Chinese and Korean immigrant parents of children 3–9 years of age [42] found that among less acculturated Chinese respondents, material deprivation was associated with more laissez-faire child feeding practices, including less monitoring, less concern about the child’s weight or diet, and less perceived responsibility for the child’s diet. Additionally, another study conducted in Maryland, U.S., with Chinese immigrant mothers of children ranging in age from 2 to 9 years found that mothers appeared to prefer ‘plumper children’ and used feeding practices such as offering their children their favorite foods [48]. Mothers who perceived their children’s body size as small and perceived their own weight and their children’s weight to be less healthy were less concerned about their children being overweight but were more likely to pressure their children to eat [48].

## 4. Discussion

This systematic review identified and synthesized the results of studies examining associations between parenting feeding styles, feeding practices, and risk of overweight and obesity among Chinese immigrant mothers of children 2–12 years of age living outside mainland China. Overall, studies included in this review found that the two most common parenting feeding styles used by Chinese mothers outside mainland China were indulgent and authoritarian [31,34,41,45,51]. Furthermore, a few studies found that children of mothers classified as having an indulgent feeding style were more likely to be overweight or obese than children of mothers classified with an authoritative feeding style [31,34]. Additionally, a longitudinal study found that feeding style was associated with nonresponisve feeding practices and child weight status. Mothers classified as having a more authoritarian parenting style were more likely to monitor child food intake, which was associated with an increased chance of having an overweight child at one-year follow-up in one study [51]. Previous studies conducted in the U.S. have demonstrated that an indulgent maternal feeding style is positively related to children’s z-BMI regardless of the mother’s race and ethnicity in pre-school-age children [53,54], and that an indulgent feeding style was associated with children’s weight status. [55]. This contrasts with studies conducted in the U.S. and the Netherlands with nonimmigrant populations that have not identified a relationship between authoritarian feeding styles and children’s weight status [53,54,55,56]. Authoritative or authoritarian feeding styles may be more commonly used in Asian populations than in other populations.

Several studies included in this review identified parenting feeding practices that have been identified in previous studies with other racial/ethnic and immigrant populations, such as restriction, pressure to eat, monitoring of food intake, and use of food as a reward and/or punishment [14,57,58]. However, the association between these unintentionally detrimental parenting feeding practices and child weight status was mixed in the reviewed studies [32,46]. For example, two studies found that restriction and use of food rewards were not associated with parent-reported child’s BMI [32,46], whereas pressuring children to eat was negatively associated with children’s weight status [46]. These findings are similar to previous studies conducted with other population groups [14,57,58]. For example, studies have found that parenting feeding practices such as restriction are negatively related to children’s weight status among school-age children in the U.S. [58] and in pre-school-age children in Australia [57]. However, lower use of restrictive feeding was found among children with lower BMI among school-age children in the U.S. [58]. In addition, a few reviewed studies identified culturally specific feeding practices commonly used in Chinese families, such as spoon-feeding and chasing after children to ensure children consume what parents perceive as a ‘healthy amount of food’ [33,52]. In traditional Chinese culture, parenting feeding practices such as spoon-feeding, chasing-after strategies, and pressuring to eat among pre-school-age children when children refuse to eat certain foods or eat too slowly are common and are culturally accepted [52]. In Chinese culture, children with a ‘healthy appetite’ and who consume large quantities of food are considered healthy [33,52].

Additionally, several reviewed studies determined that parenting feeding styles and practices varied by children’s age, weight, gender, and parents’ acculturation levels [44,45,48,49,51]. These findings are similar to prior research conducted with other minoritized and immigrant populations [59,60]. Furthermore, previous research shows that mothers are more likely to misclassify their daughters than sons as being overweight and having obesity [60]. As with previous studies, parents in the reviewed studies who reported inaccurate perceptions of their children’s weight status reported using nonresponsive child feeding practices such as control feeding, restriction, and pressure to eat [13,61].

Moreover, the findings of this review suggest that acculturation level plays an important role in parenting styles and feeding practices among Chinese immigrant families. However, only five studies [31,34,42,45,52] assessed parents’ acculturation levels, and all were conducted in the U.S. For example, one study with parents who self-identified as Chinese living in Boston, U.S. found that more acculturated parents were more likely to allow children to have sugary snacks, more likely to keep food out of pre-school children’s reach, less likely to restrict food and beverage choices, less likely to expect their pre-school-age children to finish all their food, and less likely to pressure their children to eat, although parents’ acculturation level was not related to children’s BMI [43]. In addition, a cross-sectional study conducted in New York City, U.S. found that parental higher acculturation level was negatively related to pressuring children to eat [34]. Meanwhile, parental acculturation level was positively related to monitoring children’s food consumption but not to children’s weight status [34]. In contrast, another cross-sectional study also conducted in the U.S. (Maryland) did not find any significant associations between parent acculturation level and parenting feeding practices among Chinese-American children ages three to eight [42].

Our evaluation of the methodologies of studies included in this systematic review identified some possible imitations and therefore caution in interpreting study findings. First, most of the quantitative studies reviewed (11 of 14 studies) used a cross-sectional study design. Therefore, it cannot be determined if parenting feeding styles and practices impact a child’s weight status. Second, many studies (6/15) reviewed included both mothers and fathers. Although in the Chinese culture, mothers are often the main parent in charge of cooking, meal preparation, and child feeding [51], fathers may also play a role in the types of foods served to the family and influence the family feeding environment [2,62]. Third, there was variability in how studies measured and reported children’s weight status. Some studies relied on parent-reported weight status, which may lead to misclassification of child weight status and makes it difficult to compare across studies. Finally, more than half of the included studies (9/15) were conducted in the U.S. Although the Chinese immigrant population has increased rapidly in the U.S. over the past decades, the findings of these studies may not represent those of Chinese immigrants in other countries around the world.

Despite these limitations, the current review has considerable strengths. The use of the PRISMA statement strengthens the systematic identification and assessment of eligible studies. Including qualitative, quantitative, and mixed methods studies allows for a more comprehensive understanding of the associations between parenting feeding styles and practices and the risk of overweight and obesity among Chinese children living outside mainland China. Furthermore, the use of the STROBE and CASP allowed the assessment of the quality of included studies, with the majority (n = 9) of the reviewed studies classified as strong (n = 2) [51,52] or moderate (n = 7) [44,45,46,47,48,49,50]. In addition, the reviewed studies were conducted in the past two decades, with the majority (n = 9) being conducted in the past seven years (2015–2022). Therefore, this review provides current evidence of the associations between parenting feeding styles, parenting feeding practices, and risk of overweight and obesity among Chinese children in families living outside mainland China that may be used for the design of interventions to address modifiable nonresponisve feeding styles and practices. Given the importance of parenting feeding styles and feeding practices on children’s weight status, it is likely that additional studies will be available in years to come, thus making it possible to expand on the findings of the current review.

## 5. Conclusions

In conclusion, this review summarizes the evidence from studies examining parenting feeding styles and feeding practices associated with children’s risk of being overweight or obese among children of Chinese immigrants living outside of mainland China. Although the findings of the reviewed studies suggest potentially modifiable parenting feeding styles and practices that may be addressed in interventions designed to prevent and reduce child overweight and obesity among Chinese children in families living outside mainland China, additional research is needed. Given the lack of qualitative studies in this review, future qualitative studies may help identify culturally specific feeding practices among Chinese parents living outside mainland China that may help elucidate culturally specific practices that are amenable to intervention. Furthermore, additional longitudinal studies are needed to help understand how parenting feeding styles and feeding practices influence child weight status across different stages in childhood. Finally, the majority of studies reviewed focused on mothers only. Although, as in other cultures, Chinese mothers may be the parent bearing most of the responsibility and involvement in child feeding, understanding fathers’ influence on child feeding practices and weight status would be important for developing future family interventions designed to promote healthy feeding practices.

## Figures and Tables

**Table 1:** Quality assessment of included quantitative studies using adapted ‘Strengthening the Reporting of Observational Studies in Epidemiology (STROBE)’ statement (n = 14).

Items:										
#1. Is the study longitudinal?										
#2. Does the paper describe the participants’ eligibility criteria?										
#3. Were study participants randomly selected (or representative of the study population)?										
#4. Did the paper report information about the measures, including references used to assess parental feeding practices?										
#5. Did the study include information on instrument or scale used to assess parental feeding practices that have acceptable reliability?										
#6. Did the study provide information power calculation to detect hypothesized relationships?										
#7. Did the study report the number of individuals who completed each of the different measures?										
#8. Did the participants/respondents complete at least 80% of measures?										
#9. Did analyses take into account confounding factors?										
Items										
Studies	#1	#2	#3	#4	#5	#6	#7	#8	#9	Total
Korani et al. [41]	0	1	0	1	0	1	0	0	1	4
Cheah et al. [42]	0	1	0	1	1	1	0	0	1	5
Chen et al. [31]	0	1	0	1	1	1	0	0	1	5
Chomitz et al. [43]	0	1	0	1	0	1	1	0	1	5
Huang et al. [32]	0	1	0	1	1	1	0	0	1	5
Pai et al. [34]	0	1	0	1	1	1	0	0	1	5
Chang et al. [44]	0	1	0	1	0	1	1	1	1	6
Gu et al. [45]	0	1	0	1	1	1	1	0	1	6
Liu et al. [46]	0	1	0	1	0	1	1	1	1	6
Lo et al. [47]	0	1	0	1	1	1	1	0	1	6
Vu et al. [48]	0	1	0	1	1	1	1	1	0	6
Leung et al. [49]	1	1	0	1	1	1	1	0	1	7
Sobko et al. [50]	1	1	0	1	0	1	1	1	1	7
Tung et al. [51]	1	1	0	1	1	1	1	1	1	8

**Table 2:** Methodological assessment of the qualitative study included in the integrative review (n = 1).

Qualitative Evaluation Criteria	Zhou et al. 2014 [52]
Was there a clear statement of the aims of the research?	Yes
Was the research design appropriate to address the aims of the research?	Yes
Was the recruitment strategy appropriate to the aims of the research? (e.g., How were participants selected? e.g., purposive, convenience, consecutive, snowball?; How were participants approached? e.g., face-to-face, telephone, mail, email?)	Yes
Were data collected in a way appropriate to address the research questions? (e.g., Were questions, prompts, guides provided by the authors? Was it pilot tested? Was data saturation discussed?)	Yes
Was the relationship between researcher and participants considered?	No
Were ethical issues taken into consideration?	Yes
Was the data analysis sufficiently rigorous?	Yes
Is there a clear statement of findings?	Yes
Is the research valuable?	Yes

**Table 3:** Description of studies included in the integrative review (n = 15).

Characteristics	Number of Studies
Eligible studies	15
Publication dates	
2000–2007	1
2008–2014	5
2015–2022	9
Research methods (study design)	
Qualitative	1
Quantitative (cross-sectional)	11
Quantitative (longitudinal)	3
Countries/Regions represented	
United States	9
Hong Kong	3
Australia	1
Taiwan	1
United Kingdom	1
Focus	
Feeding practices	8
Feeding practices and styles	5
Feeding styles	2

**Table 4:** Characteristics and findings of studies examining child-feeding beliefs, attitudes, knowledge, and practices of Chinese-immigrant mothers living outside China included in the integrative review (n = 15).

Authors, Year Country	Sample Characteristics and Study Design	Study Aim (s)	Measures	Main Findings Related to Parenting Feeding Styles and Practices
**Qualitative Study**				
Zhou et al. [52], 2015 U.S.	n = 22 Immigrant mothers. Mothers’ age range: 34–49 years. Children’s age range: 3–5 years. Qualitative design.	To identify whether parental feeding practices among Chinese mothers are similar to those identified in studies of European-origin families, as well as feeding practices that appear to be culturally emphasized or unique.	Focus group discussions. Open-ended questions with prompts designed to explore parental feeding practices regarding: (a) the important issues in their feeding, (b) how mothers made sure their children ate the types of foods they wanted them to eat, and (c) how mothers got their child to eat the right amount of food.	Thirteen key themes: nine known feeding practices and four culturally emphasized practices were identified. Pre-existing feeding practices included control, pressuring, restriction, use of food as reward and punishment, monitoring food intake (type and amount), and encouraging healthy eating. Culturally emphasized feeding practices included regulating healthy routines and food intake, spoon-feeding, using social comparison to pressure the child to eat, and making an effort to prepare/cook specific foods.
**Quantitative Studies**				
**Study Sample Limited to Parents of Pre-school-age Children Only**				
Liu et al. [46], 2014 Australia	n = 254 Chinese immigrant mothers who had lived in in Australia for less than 10 years. Mothers’ age range: 24–47 years. Children’s age range: 1–4 years. Cross-sectional.	To evaluate: (1) the psychometric properties and factor structure of a modified version of the Child Feeding Questionnaire (CFQ) and (2) the association between the CFQ factors in the ‘best-fitting’ model and children’s weight status.	Modified CFQ	The use of food rewards and restrictions appeared to have different influences on child health outcomes. Mothers’ feeding beliefs and perceptions of feeding responsibility were positively associated with feeding practices such as restriction, pressuring children to eat, monitoring children’s food intake, and using food rewards. Mothers’ concern about children becoming overweight was not associated with any assessed feeding practices. Mothers had high levels of perceived feeding responsibility and low levels of concern; however, these were associated with their children’s weight status. Restriction and use of food rewards were not associated with children’s weight status reported by parents. Pressuring children to eat was negatively associated with children’s weight status. Mothers who perceived their children as ‘thin’ were more likely to pressure them to eat.
Lo et al. [47], (2015) Hong Kong	n = 4553 Chinse immigrant mothers and fathers. Parents’ age is unclear. Children’s age range: 2–5 years. Cross-sectional.	To investigate the association between parental feeding styles and dietary intake among pre-school students in Hong Kong.	Parental Feeding Style Questionnaire (PFSQ)	Instrumental and emotional feeding styles were associated with unhealthy dietary patterns such as inadequate consumption of fruit, vegetables, and breakfast. They also was positively correlated with the intake of high-energy-density food. Encouraging children to eat was associated with more frequent consumption of fruits, vegetables, dairy products, and breakfast. ‘Control over child eating’ correlated with children more frequently consuming fruits, vegetables and breakfast, as well as less consumption of dairy products and high-energy-density food.
Chang et al. [44], (2017) U.S.	n = 253 Chinese immigrant mothers and fathers. Parents’ mean age: 32.7 ± 5.4 years. Children’s age range: 24–59 months. Cross-sectional.	To examine the associations between controlling feeding style, parent perception of child’s weight, and gender in Chinese families with young children.	Parent feeding style was assessed using the restriction factor (eight questions) and the pressuring factor (four questions) derived from the CFQ. Parent’s perception of child weight was assessed using a question adapted from National Health and Nutrition Examination Survey (NHANES) III. Child height and weight were obtained through review of the medical record.	Parents’ underperception of child weight was common but more likely in boys than girls. The mean pressuring and restriction scores were high, suggesting an endorsement of both styles for the overall sample. Parents were more likely to pressure boys than girls to eat. Parents’ pressure to eat did not vary significantly with their perception of their children’s weight status. However, pressure to eat was lower for girls perceived as overweight than those perceived as normal or underweight. Restrictive feeding style was not associated with parents’ perception of children’s weight, gender, or actual weight status.
Chomitz et al. [43], (2017) U.S.	n = 132 Chinese immigrant mothers and fathers. Parents’ age unclear. Children’s age range: 3.5–6.0 years. Cross-sectional.	To describe results from a community-initiated needs assessment of the eating and active living behaviors of pre-school-age Asian children in Chinatown-based early education and care programs, as well as the parenting styles of the parents/caregivers who completed the survey.	Thirteen adapted items were used to characterize three parenting practices associated with healthy eating and obesity, such as appropriate attention to hunger and satiety cues and authoritative, indulgent, or permissive styles.	Parenting practices such as control and restriction known to be associated with the risk of obesity were apparent. Although healthy-living behavioral outcomes were less prevalent among less acculturated parents, multivariable adjustment attenuated the observed significant differences.
Sobko et al. [50] (2017) Hong Kong	n = 38 Chinese immigrant mothers and their female domestic helpers. Mothers’ mean age: 36.76 ± 4.00 years. Domestic helpers’ mean age: 35.84 ± 7.26 years. Children’s age range: 2–4 years. Pilot intervention.	To test if intervention activities promote positive changes in caregivers’ feeding practices and eating habits in pre-school children.	PFSQ Hong Kong Children’s Dietary Habit Questionnaire (HKCDHQ).	Feeding practices, particularly promoting and encouraging children to eat and instrumental feeding improved after the intervention. Domestic helpers’ responsibility for children’s cooking and instrumental feeding practices predicted children’s picky eating.
Leung et al. [49] (2018) Hong Kong	n = 470 Chinese immigrant mothers and fathers. Mothers’ mean age: 34.5 ± 5.27 years. Fathers’ mean age: 38.5 ± 6.82 years. Children’s age range: 3–6 years. Longitudinal.	To examine the influence of family mealtime environment, parenting styles, and family functioning on children’s behavior.	Parenting Styles and Dimensions Questionnaire (PSDQ). Chinese Family Assessment Instrument (C-FAI). HKPFQ.	A higher frequency of child behavioral problems was associated with more authoritarian and permissive parenting styles, less healthy feeding practices, and the child being male. Family feeding practice was a mediator between permissive/authoritarian parenting and frequency of child behavior problems.
**Pre-school and School-Age Children**				
Cheah et al. [42] (2012) United States	n = 81 Chinese immigrant parents. Mothers and fathers. Mothers’ age range: 32–51 years. Fathers’ age range: 28–52 years. Children’s age range: 3–8 years. Cross-sectional.	To explore the relationships between Chinese immigrant and non-White Hispanic parents’ early life material and food deprivation and (1) concern about their child’s diet or weight; (2) preferences for plumpness; and (3) weight-promoting diet and outcomes.	CFQ	Parents’ early life food insecurity was associated with the evaluation that their child should weigh more than they do and children’s greater consumption of soda and sweets among the least acculturated parents. Parental material deprivation was associated with more laissez-faire child feeding practices: less monitoring, less concern about the child’s weight or diet, and less perceived responsibility for the child’s diet, but only among less acculturated parents. Immigrant parents’ child feeding practices and body size evaluations were shaped by material hardship in childhood, but these influences may fade as acculturation occurs.
Huang et al. [32] (2012) United States	n = 50 Chinese immigrant and Chinese-American mothers. Mothers only. Mothers’ age: unclear. Children’s age range: 2–12 years. Cross-sectional.	To gain a better understanding of attitudes, beliefs, and child feeding practices in Chinese-Americans and to explore these practices in relation to obesity risk.	CFQ	Findings determined that Chinese-American mothers had higher mean scores of concerns and restriction in all age groups and monitoring than non-Hispanic white mothers. No feeding practices were associated with child BMI in Chinese-Americanss.
Vu et al. [48], (2020) United States	n = 216 Mothers only. Mothers’ mean age: 38.31 ± 4.34 years. Children’s age range: 2.40–9.54 years. Cross-sectional.	To examine the underlying factor structure of the original CFQ (7-factor model) and the modified CFQ with additional Asian cultural-specific feeding items (eight- and nine-factor model). The validity if the CFQ among U.S. Chinese immigrant mothers also was examined.	CFQ	The nine-factor model, which included cultural-specific feeding items, was the most optimal model to represent the factor structure of feeding beliefs and practices among Chinese immigrant mothers of young children in the U.S. Mothers’ feeding beliefs and practices were associated with children’s and mothers’ BMI and mothers’ perceptions of their children’s body size.
**Study Sample Limited to Parents of School-Age Children Only**				
Chen et al. [31], (2005) United States	n = 68 Chinese immigrant mothers. Mothers’ mean age: 42.09 ± 3.81 years. Children’s age range: 8–10 years. Cross-sectional.	To examine factors associated with obesity in Chinese-American children.	Food Frequency Questionnaire (FFQ).	Three variables predicted children’s BMI, older age, a more democratic parenting style, and poor communication. Children whose mothers had a low level of acculturation were more likely to be overweight than children whose mothers were highly acculturated.
Pai et al. [34], (2014) United States	n = 712 Chinese immigrant mothers. Mothers’ age range: 25–56 years. Children’s age range: 5–10 years. Cross-sectional.	To explore the relationships between parental perceptions, feeding practices, feeding styles, parental acculturation, and child weight status.	CFQ and the Caregiver’s Feeding Styles Questionnaire (CFSQ).	Level of maternal acculturation was not directly predictive of child overweight. Chinese-American mothers tended to use indulgent (33.2%) and authoritarian (27.9%) feeding styles, with the former increasing with greater acculturation and the latter decreasing. Indulgent mothers were more likely to have more overweight and obese children, and authoritarian and authoritative parents fewer. Level of maternal acculturation was negatively predictive of pressure to eat healthy foods, which was negatively correlated with child weight status. Level of maternal acculturation was positively correlated with responsiveness to child needs, monitoring of child intake, and perceived responsibility for child feeding.
Tung et al. [51], (2014) Taiwan	n = 465 mother-child dyad. Mothers’ mean age: 37.05 ± 4.19 years for boys, 37.03 ± 4.95 years for girls. Children’s mean age: 8.45 ± 1.03 years for boys, 8.35 ± 1.02 years for girls at baseline. Longitudinal.	To examine the associations between child feeding practices and weight status changes over 1 year among a sample of school-age children in Taiwan.	PSDQ CFQ	Controlling for baseline weight status revealed moderating effects of parenting style on the relationship between child feeding practices and child weight status. Two feeding practices, concerning child weight and monitoring, were significant predictors of child overweight status among children with authoritative mothers. Mothers with a stronger perceived responsibility for child feeding also scored higher on pressure to eat and monitor. Mothers who were concerned about their daughter’s weight status employes feeding practices involving more restriction of food and less pressure to eat. Parents’ perpetion of their children’s weight and concern about child weight increased from children’s underweight to obesity. Parents with underweight children used more pressure to eat than those with overweight children.
Korani et al. [41], (2018) United Kingdom	n = 84 Chinese immigrant mothers. Mothers only. Mother’s age range: 23–54 years. Children’s age range: 5–11 years. Cross-sectional.	To explore variations in maternal child-feeding style between ethnic groups in the U.K., considering associated factors such as deprivation and parenting style.	CFQ PFSQ PSDQ	Significant differences in perceived responsibility, restriction, pressure to eat, instrumental feeding, and emotional feeding were found between the groups. Mothers from Chinese backgrounds reported greater perceived responsibility and use of restriction. Maternal child-feeding style was associated with deprivation and parenting style.
Gu et al. [45], (2022) United States	n = 233 Chinese-American parents. Mothers and fathers. Parents’ mean age: 42.4 ± 5.5 years. Children’s age range: 5–12 years. Cross-sectional.	To investigate associations of acculturation with parents’ food-related parenting and general parenting behaviors in a sample of predominantly first-generation immigrant Chinese-American parents of school-age children. To test associations of parent feeding and general parenting behaviors with child body mass index *z*-score in this sample, based on parent-report data of child height and weight.	CFQ PFSQ CFSQ PSDQ	Acculturation was associated with higher scores on responsiveness in feeding, lower scores on subscales assessing controlling feeding behaviors, lower scores on nonnutritive feeding behaviors, and a greater likelihood of indulgent feeding styles. Acculturation was associated with lower scores on subscales assessing authoritarian parenting. Parental prompting/encouragement to eat was associated with a lower child BMI *z*-score, while authoritarian parenting subscales were associated with a higher BMI *z*-score.

## Data Availability

Data used in this review were available in the articles included in this review which are available in the following databases CINAHL, Medline, PsycINFO, and PubMed.

## Supplementary Materials

The following supporting information can be downloaded at: https://www.mdpi.com/article/10.3390/ijerph20054090/s1, Figure S1: PRISMA flow diagram, Table S1: Quality assessment of included quantitative studies using adapted ‘Strengthening the Reporting of Observational Studies in Epidemiology (STROBE) statement; Table S2: Quality assessment of included qualitative studies using the Critical Appraisal Skills Program (CASP); Table S3: Description of studies included in systematic review; Table S4: Characteristics of studies examining infant feeding beliefs, attitudes, knowledge and practices of Chinese immigrant mothers included in integrative review.
