# Supplementary material for: Nonresponsive Parenting Feeding Styles and Practices and Risk of Overweight and Obesity among Chinese Children Living Outside Mainland China: An Integrative Review of the Literature
Source: Int J Environ Res Public Health. 2023 Feb 24;20(5):4090. doi: 10.3390/ijerph20054090 (PMC10002266; doi:10.3390/ijerph20054090)
Supplement: Supplementary file 1 [file ijerph-20-04090-s001.zip › ijerph-2164753-supplementary.pdf]

Figure S1: PRISMA 2009 flow diagram*

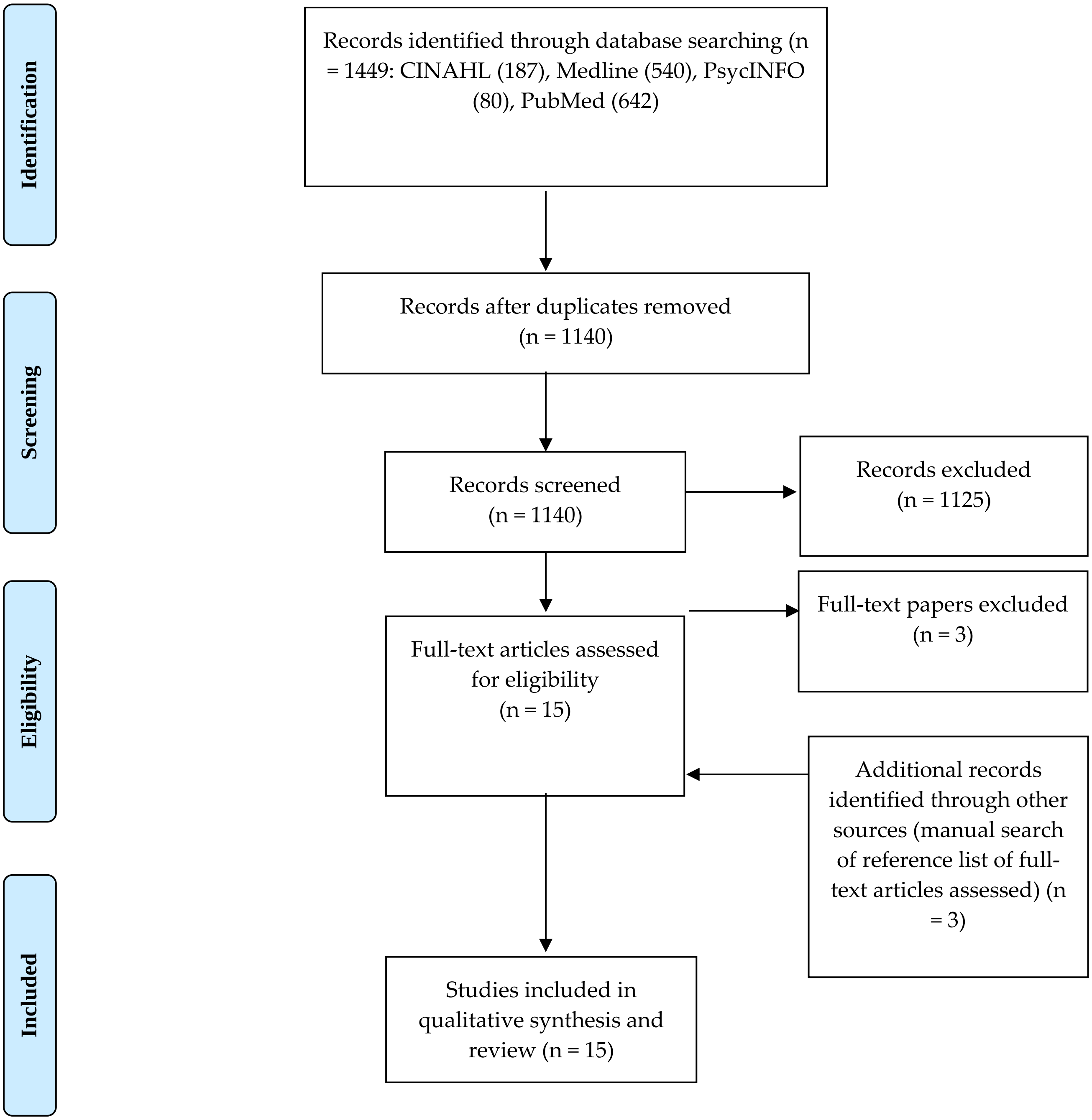

*From: Moher D, Liberati A, Tetzlaff J, Altman DG, The PRISMA Group (2009). *P*referred *R*eporting *I*tems for *S*ystematic Reviews and *M*eta-*A*nalyses: The PRISMA Statement. PLoS Med 6(7): e1000097. doi:10.1371/journal.pmed1000097

Table S1. Quality assessment of included quantitative studies using adapted "Strengthening the Reporting of Observational Studies in Epidemiology (STROBE)" statement (n=14)

Items:

#1. Is the study longitudinal?
#2. Does the paper describe the participants' eligibility criteria?
#3. Were study participants randomly selected (or representative of the study population)?
#4. Did the paper report information about the measures, including references used to assess parental feeding practices?
#5. Did the study include information on instrument or scale used to assess parental feeding practices that have acceptable reliability?
#6. Did the study provide information power calculation to detect hypothesized relationships?
#7. Did the study report the number of individuals who completed each of the different measures?
#8. Did the participants/respondents complete at least 80% of measures?
#9. Did analyses take into account confounding factors?

	Items									
**Studies**	**#1**	**#2**	**#3**	**#4**	**#5**	**#6**	**#7**	**#8**	**#9**	**Total**
Korani et al. [43]	0	1	0	1	0	1	0	0	1	4
Cheah et al. [40]	0	1	0	1	1	1	0	0	1	5
Chen et al. [29]	0	1	0	1	1	1	0	0	1	5
Chomitz et al. [41]	0	1	0	1	0	1	1	0	1	5
Huang et al. [30]	0	1	0	1	1	1	0	0	1	5
Pai et al. [32]	0	1	0	1	1	1	0	0	1	5
Chang et al. [39]	0	1	0	1	0	1	1	1	1	6
Gu et al. [42]	0	1	0	1	1	1	1	0	1	6
Liu et al. [45]	0	1	0	1	0	1	1	1	1	6
Lo et al. [46]	0	1	0	1	1	1	1	0	1	6
Vu et al. [49]	0	1	0	1	1	1	1	1	0	6
Leung et al. [44]	1	1	0	1	1	1	1	0	1	7
Sobko et al. [47]	1	1	0	1	0	1	1	1	1	7
Tung et al. [48]	1	1	0	1	1	1	1	1	1	8

Table S2. Methodological assessment of qualitative studies included in integrative review (n=1).

Qualitative Evaluation Criteria	Zhou et al. 2014 [50]
Was there a clear statement of the aims of the research?	Y
Was the research design appropriate to address the aims of the research?	Y
Was the recruitment strategy appropriate to the aims of the research? (e.g., How were participants selected? e.g., purposive, convenience, consecutive, snowball?; How were participants approached? e.g., face-to-face, telephone, mail, email?)	Y
Were data collected in a way appropriate to address the research questions? (e.g., Were questions, prompts, guides provided by the authors? Was it pilot tested? Was data saturation discussed?)	Y
Has the relationship between researcher and participants been considered?	N
Have ethical issues been taken into consideration?	Y
Was the data analysis sufficiently rigorous?	Y
Is there a clear statement of findings?	Y
Is the research valuable?	Y

Y=yes, N=no, U=uncertain.

Table S3. Description of studies included in integrative review (n=15)

Characteristics	No. of Studies
**Total number of studies selected**	15
**Publication dates**	
2000 – 2007	1
2008 – 2014	5
2015 – 2022	9
**Research Methods (study design)**	
Qualitative	1
Quantitative (cross-sectional)	11
Quantitative (longitudinal)	3
**Countries/Regions represented**	
United States	9
Hong Kong	3
Australia	1
Taiwan	1
United Kingdom	1
**Feeding practices and styles**	
Feeding practices	8
Feeding practices and styles	5
Feeding styles	2
**Assessment of child feeding practices**	
Open-ended interviews	1
The Child Feeding Questionnaire (CFQ)	9
Parental Feeding Styles Questionnaire (PFSQ)	4
Parenting Styles and Dimensions Questionnaire (PSDQ)	4
Caregiver's Feeding Styles Questionnaire (CFSQ)	2
Other (i.e., ATCRS, FAD, FFQ, HKCDHQ, HKPFQ)	7

Table S4. Characteristics and findings of studies examining child feeding beliefs, attitudes, knowledge, and practices of Chinese-immigrant mothers living outside China included in integrative review (n = 15).

Authors, Year Country	Sample characteristics and study design	Study Aim (s)	Measures of infant and/or child feeding practices	Main Findings
**Qualitative Study**				
Zhou et al. [50], 2015 United States	n = 22 (immigrant mothers)<br><br>Mothers only. Mothers' age range: 34-49 years. Children's age range: 3-5 years.<br><br>Qualitative design	To identify whether parental feeding practices among Chinese mothers that are similar to feeding practices that have already been identified in studies of European-origin families, as well as feeding practices that appear to be culturally emphasized or unique.	Focus group discussions. Focus group questions were developed based on a review of feeding questionnaires in the literature by a panel (two developmental psychologists, one sociologist/demographer, and one pediatrician) with joint expertise in parenting and obesity across various ethnic groups.<br><br>Open-ended questions with prompts generated discussions on parental feeding practices regarding: (a) the important issues in their feeding, (b) how mothers made sure their child ate the types of foods they wanted them to eat, and (c) how mothers got their child to eat the right amount of food.<br><br>Demographic questionnaire.	Thirteen key themes including 9 pre-exiting feeding practices, and 4 culturally emphasized practices were identified.<br><br>Pre-existing feeding practices included: control, pressuring, and restriction, use of food as reward and punishment, monitoring of food intake (type and amount), and encouraging healthy eating.<br><br>Culturally emphasized feeding practices included: regulating healthy routines and food intake, spoon-feeding, using social comparison to pressure the child to eat, and making an effort to prepare/cook specific foods.

**Quantitative Studies**				
**Preschool-age Children Only**				
Liu et al. [45], 2014<br>Australia	n = 254 Chinese immigrant mothers living less than 10 years in Australia.<br><br>Mothers only. Mothers' age range: 24-47 years. Children's age range: 1-4 years.<br><br>Cross-sectional	Two-fold objectives: 1) to evaluate the psychometric properties and factor structure of a modified version of the CFQ with a sample of Chinese mothers of young children in Australia and, 2) to examine the association between the CFQ factors in the 'best-fitting' model and children's weight status.	Modified CFQ.	The modified eight-factor CFQ appeared to be a linguistically and culturally appropriate instrument for assessing feeding beliefs and practices in Chinese-Australian mothers of young children.<br><br>The eight-factor structure in which the factors use of food rewards and restriction were separated was argued to be the best-fitting model in this sample.<br><br>Use of food rewards and restriction appeared to have different influences on child health outcomes.<br><br>Mothers' feeding beliefs and perceptions of feeding responsibility were positively associated with their use of feeding practices such as restriction, pressuring children to eat, monitoring of children's food intake and use of food rewards.<br><br>Mothers' concern about children becoming overweight was not associated with any of the feeding practices.<br><br>High levels of perceived feeding responsibility and low levels of concern were demonstrated among the mothers. Neither of these feeding beliefs were found to be associated with their children's weight status.<br><br>Restriction and use of food rewards were not associated with parent-reported child's BMI.<br><br>Pressuring children to eat was shown to be negatively associated with children's weight status. Mothers who perceived their children as being "thin" were more likely to pressure their children to eat.

Lo et al. [45], (2015) Hong Kong	n = 4553<br><br>Mothers and fathers. Parents' age unclear. Children's age range: 2-5 years.<br><br>Cross-sectional	To investigate the association between parental feeding styles and dietary intake among pre-school students in Hong Kong.	Parental Feeding Style Questionnaire (PFSQ)<br><br>Sociodemographic and dietary intake questionnaire.	Instrumental and emotional feeding was associated with unhealthy dietary pattern. Instrumental and/or emotional feeding was found to relate to inadequate consumption of fruit, vegetables, and breakfast, and positively correlated with intake of high-energy-density food.<br><br>"Encouragement child eating" was associated with more frequent consumption of fruits, vegetables, dairy products, and breakfast.<br><br>"Control over child eating" correlated with more frequent consumption of fruits, vegetables and breakfast, and less consumption of dairy products and high-energy-density food.
Chang et al. [39], (2017) United States	n = 253<br><br>Mothers and fathers. Parents' mean age: 32.7±5.4 years. Children's age range: 24-59 months.<br><br>Cross-sectional	To examine the associations between controlling feeding style, parent perception of weight, and gender in Chinese American families with young children.	Parent feeding style was assessed using the restriction factor (eight questions) and the pressuring factor (4 questions) derived from the Child Feeding Questionnaire (CFQ), a validated self-report measure to assess parental beliefs, attitudes, and practices regarding child feeding.<br><br>Parent Perception of Child Weight was assessed by question adapted from National Health and Nutrition Examination Survey (NHANES) III.	Parent under-perception of weight was common but more likely in boys than girls.<br><br>Both the mean pressuring and restriction scores were high suggesting endorsement of both styles for the overall sample. Pressuring was significantly higher in boys in both unadjusted and adjusted analyses.<br><br>In boys, pressuring to eat was higher overall, and did not vary significantly with perception of weight status, while in girls, pressuring to eat was lower for girls perceived as overweight compared to those perceived as normal or underweight.<br><br>Restrictive feeding style was not associated with perception of weight, gender, or actual weight status

			Child height and weight, which were obtained by review of the medical record.	
Chomitz et al. [41], (2017)<br>United States	n = 132<br><br>Mothers and fathers. Parents' age unclear. Children's age range: 3.5-6.0 years.<br><br>Cross-sectional	To describe results from a community-initiated needs assessment of the eating and active living behaviors of pre-school aged Asian children, in Chinatown-based early education and care programs, as well as the parenting styles of the parents/ caregivers who completed the survey.	Healthy food and beverage outcomes assessed via frequency of consumption of food.<br><br>Thirteen adapted items were used to characterize three domains of parenting practices that have been associated with healthy eating and obesity, such as appropriate attention to hunger and satiety cues; authoritative; and indulgent or permissive.	Parenting practices such as control and restriction known to be associated with risk of obesity were apparent.<br><br>Although healthy-living behavioral outcomes were less prevalent among less acculturated parents; multivariable adjustment attenuated the observed significant differences.
Sobko et al. [46] (2017)<br>Hong Kong	n = 38 mothers and their female domestic helpers.<br><br>Mothers' mean age: 36.76±4.00 years.<br><br>Domestic helpers' mean age: 35.84±7.26 years.<br><br>Children's age range: 2-4 years.<br><br>Pilot intervention	To test the intervention activities whether induce positive changes in caregivers' feeding practices and eating habits in preschool children.	Parental Feeding Style Questionnaire (PFSQ).<br><br>Hong Kong Children's Dietary Habit Questionnaire. (HKCDHQ)	Feeding practices, particularly promoting, and encouraging children to eat and instrumental feeding improved after the intervention.<br><br>Mother's BMI, responsibility for child's meal, child's birth weight had a bearing on the improvement of "promoting and encouragement to eat".<br><br>Domestic helper's responsibility for child's cooking and instrumental feeding practices predicted child's picky eating.<br><br>Mother's responsibility for child and helper's responsibility for cooking predicted child's consumption of salty foods.

Leung et al. [44] (2018)<br>Hong Kong	n = 470<br><br>Mothers and fathers.<br>Mothers' mean age: 34.5±5.27 years.<br>Fathers' mean age: 38.5±6.82 years.<br><br>Children's age range: 3-6 years<br><br>Longitudinal	To examine the influence of family mealtime environment, parenting styles, and family functioning on children's behavior.	Parenting Styles and Dimensions Questionnaire (PSDQ).<br><br>Chinese Family Assessment Instrument (C-FAI)<br><br>Hong Kong Parent Feeding Questionnaire (HKPFQ)<br><br>Family Meal Frequency<br><br>Frequency of mealtime television viewing<br><br>Eyberg Child Behavior Inventory	Higher frequency of child behavior problems was associated with more authoritarian and permissive parenting styles, less healthy feeding practice, and the child being male.<br><br>Family feeding practice was a mediator between permissive/authoritarian parenting and frequency of child behavior problems.
**Preschool and School-Age Children**				
Cheah et al. [40] (2012)<br>United States	n = 81 Chinese immigrant parents.<br><br>Mothers and fathers.<br>Mothers' age range: 32-51 years. Fathers' age range: 28-52 years.<br><br>Children's age range: 3-8 years<br><br>Cross-sectional	To explore the relationships between immigrant parents' early life material and food deprivation and (1) concern about their child's diet or weight; (2) preferences for plumpness; (3) weight-promoting diet and outcomes.	Child Feeding Questionnaire (CFQ).<br><br>Parent's early life deprivation.<br><br>Chinese Children Acculturation Scale.<br><br>The modified Stunkard Body Rating Scale adapted for children.	Parent's early life food insecurity was associated with the evaluation that their child should weigh more than they do and greater consumption of soda and sweets by their child, among the least acculturated parents.<br><br>Parental material deprivation was associated with more laissez-faire child feeding practices: less monitoring, less concern about the child's weight or diet, and less perceived responsibility for child's diet, but only among less acculturated parents.<br><br>Immigrant parent's child feeding practices and body size evaluations were shaped by material hardship in childhood, but these influences may fade as acculturation occurs.

Huang et al. [30] (2012) United States	n = 50 Chinese American mothers.<br><br>Mothers only.<br>Mothers' age: unclear.<br>Children's age range: 2-12 years<br><br>Cross-sectional	To gain a better understanding of attitudes, belief, and child-feeding practices in Chinese Americans and explore these practices in relation to obesity risk.	Child Feeding Questionnaire (CFQ).	Findings determined that Chinese American mothers had higher mean scores of concerns and restriction in all age groups and monitoring than non-Hispanic white mothers.<br><br>None of the feeding practices were found to be associated with child BMI in Chinese Americans.
Vu et al. [49], (2020) United States	N=216<br><br>Mothers only.<br>Mothers' mean age: 38.31 ± 4.34 years.<br><br>Children's age range: 2.40-9.54 years.<br><br>Cross-sectional	To examine the underlying factor structure of the original CFQ (7-factor model) and the modified CFQ with additional Asian cultural-specific feeding items (8- and 9-factor model) and assessed the validity of the CFQ among U.S. Chinese immigrant mothers.	Child Feeding Questionnaire (CFQ).	The 9-factor model, which included the cultural-specific feeding items, was the most optimal model to represent the factor structure of feeding beliefs and practices among U.S. Chinese immigrant mothers of young children.<br><br>Mothers' feeding beliefs and practices were associated with children's and mothers' body mass index and mothers' perceptions of their children's body size.

School-Age Children Only				
Chen et al. [29], (2005) United States	n = 68 immigrant mothers<br><br>Mothers' mean age: 42.09 ± 3.81 years.<br><br>Children's age range: 8-10 years.<br><br>Cross-sectional	To examine factors associated with obesity in Chinese American children.	Family Assessment Device (FAD).<br><br>Attitudes Toward Child-Rearing Scale (ATCRS).<br><br>Food Frequency Questionnaire (FFQ).<br><br>The Schoolagers' Coping Strategies Inventory (SCSI).	Three variables were identified predicting children's body mass index: older age, a more democratic parenting style, and poor communication.<br><br>Children whose mothers had a low level of acculturation were also more likely to be overweight than were children whose mothers were highly acculturated.
Pai et al. [32], (2014) United States	n = 712 mothers<br><br>Mothers' age range: 25-56 years. Children's age range: 5-10 years.<br><br>Cross-sectional	To explore the relationships between parental perceptions, feeding practices, feeding styles, level of parental acculturation, and child weight status via a self-administered questionnaire.	The Suinn-Lew Asian Self-Identity Acculturation scale (SL-ASIA).<br><br>Child Feeding Questionnaire (CFQ).<br><br>Caregiver's Feeding Styles Questionnaire (CFSQ).	Level of maternal acculturation was not directly predictive of child overweight in multiple regression but from categorical data, Chinese American mothers tended to use indulgent (33.2%) and authoritarian (27.9%) feeding styles, with the former increasing with acculturation and the latter decreasing.<br><br>Indulgent mothers had more than expected overweight and obese children, and authoritarian and authoritative parents, fewer.<br><br>Level of maternal acculturation was negatively predictive of pressure to eat healthy foods, which was negatively correlated with child weight status.<br><br>Level of maternal acculturation was also independently positively correlated to responsiveness to child needs, monitoring of child intake, and perceived responsibility for child feeding.

Tung et al. [48], (2014) Taiwan	n = 465 mother-child dyad<br><br>Mothers' mean age: 37.05±4.19 years for boys, 37.03±4.95 years for girls.<br><br>Children's mean age: 8.45±1.03 years for boys, 8.35±1.02 years for girls at baseline.<br><br>Longitudinal	To exam the associations between child-feeding practices and weight status changes over 1 year among a sample of school-aged children in Taiwan.	Parenting Styles and Dimension Questionnaire (PSDQ).<br><br>Child Feeding Questionnaire (CFQ).	Controlling for baseline weight status revealed moderating effects of parenting style on the relationship between child-feeding practices and child weight status.<br><br>Both authoritative and authoritarian mothers might monitor their children's dietary intake, however, the effectiveness of this practice was better, in terms of weight status control, among the authoritative mothers.<br><br>Mothers who had a stronger perceived responsibility for child feeding also scored higher on pressure to eat and monitoring.<br><br>Among the girls, if a mother was concerned about her daughter's weight status, her feeding practices would involve more restriction to eat and less pressure to eat. However, for the boys, none of these practices were significant strategies used by their mothers.<br><br>Parents' perceived child weight and concern about child weight increased from underweight to obese, while parents with underweight children used more pressure to eat than those with overweight children.

Korani et al. [43], (2018) United Kingdom	n = 84 Chinese mothers<br><br>Mothers only. Mother's age range: 23-54 years. Children's age range: 5-11 years<br><br>Cross-sectional	To explore variations in maternal child-feeding style between ethnic groups in the UK, considering associated factors such as deprivation and parenting style.	Child Feeding Questionnaire (CFQ).<br><br>Parental Feeding Styles Questionnaire (PFSQ).<br><br>Parenting Styles and Dimensions Questionnaire (PSDQ).	Significant differences in perceived responsibility, restriction, pressure to eat, instrumental feeding and emotional feeding were found between the groups.<br><br>Mothers from Chinese backgrounds reported greater perceived responsibility and restriction.<br><br>Maternal child-feeding style was also associated with deprivation and parenting style, although these did not fully explain the data.
Gu et al. [42], (2022) United States	n = 233 Chinese parents<br><br>Mothers and fathers. Parents' mean age: 42.4 ± 5.5 years. Children's age range: 5-12 years<br><br>Cross-sectional	To investigate associations of acculturation with parents' food-related parenting and general parenting behaviors in a sample of predominantly first-generation immigrant Chinese American parents of school0aged children. To test associations of parent feeding and general parenting behaviors with child body mass index z-score in this sample, based on parent-report data of child height and weight.	The Suinn-Lew Asian Self-Identity Acculturation scale (SL-ASIA).<br><br>Child Feeding Questionnaire (CFQ).<br><br>Parental Feeding Styles Questionnaire (PFSQ).<br><br>Caregiver's Feeding Styles Questionnaire (CFSQ).<br><br>Parenting Styles and Dimensions Questionnaire (PSDQ).	Acculturation was associated with higher scores on Responsiveness in feeding, lower scores on subscales assessing controlling feeding behaviors, lower scores on non-nutritive feeding behaviors, and greater likelihood of an indulgent feeding styles.<br><br>Acculturation was associated with lower scores on subscales assessing authoritarian parenting.<br><br>Parental Prompting/Encouragement to eat was associated with lower child BMI z-score, while authoritarian parenting subscales were associated with higher BMI z-score.
